# An Explanation Based on Energy-Related Changes for Blue Mussel *Mytilus edulis* Coping With Seawater Acidification

**DOI:** 10.3389/fphys.2021.761117

**Published:** 2021-10-14

**Authors:** Ying Guo, Bin Zhou, Tianli Sun, Yaya Zhang, Yongshun Jiang, You Wang

**Affiliations:** ^1^College of Marine Life Sciences, Ocean University of China, Qingdao, China; ^2^Laboratory for Marine Ecology and Environmental Science, Pilot National Laboratory for Marine Science and Technology, Qingdao, China; ^3^National Marine Hazard Mitigation Service, Beijing, China; ^4^Marine Science and Engineering College, Qingdao Agricultural University, Qingdao, China

**Keywords:** energy allocation, total adenylate pool, energy charge, adenosine triphosphate, cytochrome C oxidase, *Mytilus edulis*, seawater acidification

## Abstract

As ocean acidification (OA) is gradually increasing, concerns regarding its ecological impacts on marine organisms are growing. Our previous studies have shown that seawater acidification exerted adverse effects on physiological processes of the blue mussel *Mytilus edulis*, and the aim of the present study was to obtain energy-related evidence to verify and explain our previous findings. Thus, the same acidification system (pH: 7.7 or 7.1; acidification method: HCl addition or CO_2_ enrichment; experimental period: 21d) was set up, and the energy-related changes were assessed. The results showed that the energy charge (EC) and the gene expressions of cytochrome C oxidase (COX) reflecting the ATP synthesis rate increased significantly after acidification treatments. What’s more, the mussels exposed to acidification allocated more energy to gills and hemocytes. However, the total adenylate pool (TAP) and the final adenosine triphosphate (ATP) in *M. edulis* decreased significantly, especially in CO_2_ treatment group at pH 7.1. It was interesting to note that, TAP, ATP, and COXs gene expressions in CO_2_ treatment groups were all significantly lower than that in HCl treatment groups at the same pH, verifying that CO_2_-induced acidification exhibited more deleterious impacts on *M. edulis*, and ions besides H^+^ produced by CO_2_ dissolution were possible causes. In conclusion, energy-related changes in *M. edulis* responded actively to seawater acidification and varied with different acidification conditions, while the constraints they had at higher acidification levels suggest that *M. edulis* will have a limited tolerance to increasing OA in the future.

## Introduction

Oceans have absorbed a large amount of anthropogenic carbon dioxide (CO_2_) since the industrial revolution, leading to ocean acidification (OA; Caldeira and Wickett, 2003; Doney et al., 2009). The average seawater surface pH has declined by approximately 0.1units compared with pre-industrial levels, and it is predicted to decline by an additional 0.3–0.4units by the end of this century (Fabry et al., 2008; Gattuso et al., 2015). OA has been reported to exerted adverse effects on marine organisms, including fertilization (Shi et al., 2017; Han et al., 2021), physiological responses (Peng et al., 2017; Zhao et al., 2017a,b, 2020), immune responses (Liu et al., 2016; Su et al., 2018), behavioral responses (Peng et al., 2017; Rong et al., 2018, 2020), and so on (Liu, 2021). Kroeker et al. (2011) findings indicated that OA decreased the diversity, biomass, and trophic complexity of benthic marine communities in reduced pH zones, with the highest reduction seen in the key calcifying species. In fact, natural CO_2_ vents in the Mediterranean Sea and Indo-Pacific are virtually devoid of calcifying organisms at pH 7.7 and below (Hall-Spencer et al., 2008). Owing to relatively low metabolic rates, poor acid-base regulation capacities and calcareous skeletal structures or shells, a large fraction of mollusk species presented a high vulnerability to OA (Thomsen et al., 2010; Wittmann and Pörtner, 2013; Wang and Wang, 2020). Bivalve mollusks, particularly in the early stages of their life, can react with decreased rates of growth and calcification, as well as a decreased shell strength toward elevated seawater *p*CO_2_ (Beniash et al., 2010; Gazeau et al., 2010; Talmage and Gobler, 2010; Gaylord et al., 2011; Stevens and Gobler, 2018). The slowness of growth and calcification seen in bivalve mollusks under acidic conditions was attributed to a higher energy consumption for the maintenance of physiological homeostasis (Lannig et al., 2010; Thomsen and Melzner, 2010; Melzner et al., 2020). For example, the total energy loss of *Mytilus edulis* in the treatments at pH 7.7 and pH 7.4 increased by 42 and 59%, respectively, compared with that of the control at pH 8.0 (Thomsen and Melzner, 2010). Similarly, Beniash et al. (2010) found that *Crassostrea virginica* greatly increased its energy consumption and standard metabolic rate to maintain its internal stability when it was exposed to an acidic environment at pH 7.5.

It is well documented that the organisms adopt different energy strategies when facing different acidic conditions. Organisms may compensate for the elevated energy demand during moderate acidification stress by increasing their energy intake, assimilation, and/or metabolic flux to cover the excessive demand for adenosine triphosphate (ATP). However, such compensation might be incomplete or impossible during extreme stress, and organisms would enter a metabolically depressed state to conserve energy and extend their survival time (Guppy and Withers, 1999). Melzner et al. (2011) found that strong *p*CO_2_ stress (pH < 7.4) coupled with food limitation caused *M. edulis* to prefer allocating its resources toward the conservation of somatic mass, and the nacre was partially sacrificed because it required an extra energy input to maintain an intact nacre surface in the corrosive fluid. A recent study demonstrated that oysters *Crassostrea gigas* exhibited energy modulations with slight inhibition of aerobic metabolism, stimulation of anaerobic metabolism, and an increase in the glycolytic enzyme activity after exposure to seawater acidification (Cao et al., 2018). However, another earlier report declared that the long-term acclimation of *M. edulis* to elevated seawater *p*CO_2_ in the Western Baltic Sea resulted in an increase in aerobic metabolic rates rather than metabolic depression during moderate hypercapnia (pH > 7.4; Thomsen and Melzner, 2010). It seemed that the energy responses of bivalves to seawater acidification were species-specific and varied under different acidic conditions.

Our previous studies showed that seawater acidification had adverse effects on *M. edulis* (Sun et al., 2016, 2017; Xu et al., 2020). The mortality rate of mussels increased significantly, while the rates of growth and calcification decreased considerably after acidification treatments. Ultrastructural impairments in gills, digestive glands, and hemocytes occurred, and the corresponding key physiological processes of ingestion, digestion, and immune functions were obviously influenced. What interested us most were the specific energy responses of *M. edulis* to acidified seawater and the consequent influences on *M. edulis* to cope with seawater acidification. Therefore, the present study focused on evaluating the energy responses of *M. edulis* to different acidification conditions. Understanding the energy strategies that *M. edulis* employs to address acidification stress and their biological limitations can serve as a quantitative basis for assessing the sublethal effects of seawater acidification on *M. edulis* and contribute to the prediction of the mussel population fate under future OA.

## Materials and Methods

### Organism Collection and Acclimation

Adult individuals of *M. edulis* (shell length 48.88±0.72mm and wet weight 7.20±0.58g) were collected in Laoshan Bay (36°15ʹN, 120°40ʹE), Qingdao, China, and directly transferred to the experimental aquaria. The individuals were cleaned carefully and then acclimated in the experimental tanks (Vol.=8l; 30 mussels per tank) for a week before the experiments under the controlled conditions:temperature 15±1°C; salinity 31±1; pH 8.1±0.1 (Sun et al., 2016, 2017). Natural seawater was filtered on a 0.45μm pore size membrane and completely renewed every day. Food algae, green microalgae *Chlorella* sp., was supplied daily to the holding tanks by natural gravity (approximately 1mlmin^−1^), and the final density in each tank was 1.5×10^5^cellsml^−1^.

### Experimental Design and Acidification System Setup

The acidification system consisted of two acidification levels, one representing the near-future OA (pH 7.7, *p*CO_2_≈1,500ppm; Orr et al., 2005) and the other mimicking the CO_2_ sequestration leak scenarios (pH 7.1, *p*CO_2_≈5,000ppm; Berge et al., 2006). Each acidification level was simulated by two different acidification methods of HCl addition (seawater was acidified by adding 1M HCl) and CO_2_ enrichment (seawater was bubbled with pure CO_2_ gas) according to our previous study (Sun et al., 2017). Seawater pH_NBS_ was measured and adjusted by using a pH controller (pH/ORP-101, HOTEC, Taiwan), and the pH fluctuations were controlled within 0.08units. The pH of the control was equal to the current average ocean surface value (pH 8.1, *p*CO_2_≈390ppm). The pH_NBS_ and salinity were monitored daily, while the total alkalinity (AT) was measured weekly. All the other carbonate system variables were calculated using CO2SYS software according to Pierrot et al. (2006). The chemical parameters of the seawater acidified by CO_2_ were as follows (Table 1). The acclimated mussels were randomly divided into 5 groups, including 1 control group (pH 8.1) and 4 acidifying treatment groups (2 CO_2_-treated groups: pH 7.7 and pH 7.1; 2 HCl-treated groups: pH 7.7 and pH 7.1). Each group had three parallel groups with 15 individuals in. The experiments lasted for 21days. During the experimental period, the temperature was kept at 15±1°C and the salinity of the experimental seawater was 31±1. The green microalgae *Chlorella* sp. was provided once a day at a final density of 1.5×10^5^cellsml^−1^.

**Table 1:** Key parameters (means±SD, *n*=3) of carbonate chemistry in CO_2_ enriched seawater of different pH levels, including setting seawater pH, measured pH, total alkalinity (TA), concentrations of bicarbonate (HCO_3_^−^), carbonate ion (CO_3_^2−^), carbonic acid (H_2_CO_3_), and partial CO_2_ pressure (*p*CO_2_), calculated saturation states of aragonite (Ω_Ag_), and calcite (Ω_Cal_).

pH	pH	TA	HCO_3_^−^	CO_3_^2−^	H_2_CO_3_	*p*CO_2_	Ω_Ag_	Ω_Cal_
(set)	(measured)	(μM)	(mM)	(mM)	(mM)	(μatm)		
8.1	8.10 ± 0.05	2098 ± 89	1733 ± 79	145 ± 15	14 ± 1	448 ± 39	2.25 ± 0.25	3.47 ± 0.32
7.7	7.67 ± 0.07	2098 ± 82	1946 ± 89	60 ± 8	44 ± 5	1,353 ± 107	0.94 ± 0.17	1.45 ± 0.26
7.1	7.06 ± 0.06	2098 ± 77	2059 ± 74	16 ± 2	188 ± 19	5,831 ± 321	0.24 ± 0.03	0.38 ± 0.06

### Assessments of Energy Changes

#### Sample Preparation

Six mussels in each parallel group were randomly collected on the 21st day of the experiment. The gills and soft tissue (the whole tissues except shell) were sampled and then ground with phosphate-buffered saline (PBS, 0.14M sodium chloride, 3mm potassium chloride, 8mm disodium hydrogen phosphate dodecahydrate, and 1.5mm potassium phosphate monobasic; pH 7.4), and the hemocytes were sampled according to Sun et al. (2017). The collected samples were sonicated on ice bath, and the homogenates were mixed with 20% (v/v) perchloric acid at 1:1 (v/v) and then centrifuged at 4000g, 4°C for 10min. The supernatant was adjusted to pH 6.5 with 1M KOH and added to 10ml with 10% (v/v) perchloric acid, of which the pH was preset to 6.5 with 1M KOH, and then filtered with a 0.22μm microporous membrane. A portion of the filtrate (200μl) was used for reversed-phase high-performance liquid chromatography analysis, and the remains were used for the quantitative analysis of the total proteins.

#### Measurements of the Energy Changes in Gills, Soft Tissue and Hemocytes

The total adenylate pool (TAP) is generally stable in mitochondria, and its content reflects not only the cell ability to produce high-energy phosphate compounds but also the cell energy reserve status (Xu et al., 2005). The energy charge (EC) was defined as the number of high-energy phosphate groups loaded in the total adenylate system, and it was proposed as a metabolic regulatory parameter reflecting the states of ATP regeneration and ATP utilization (Atkinson, 1968). TAP and EC could be calculated according to the following equations (Atkinson, 1968):


TAP=ATP+ADP+AMP

$$\mathrm{EC}=\left(\mathrm{ATP}+0.\mathrm{5ADP}\right)/\left(\mathrm{ATP}+\mathrm{ADP}+\mathrm{AMP}\right)$$

The adenylate (ATP, ADP, and AMP) contents of the samples were measured according to the methods of Zhang et al. (2016) and Zhu et al. (2017) with some minor adjustments. We used ultrapure water instead of phosphate buffer as the solvent and 10% (v/v) perchloric acid at pH 6.5 for the preparation of the adenylate standards, adjusted the flow rate to 0.7mlmin^−1^ and maintained the column temperature at 20°C during the analysis. The total proteins of the samples were determined by the Bradford (1976) method, and the ATP, ADP, and AMP contents were normalized to their corresponding total proteins.

### qRT-PCR Analysis of COXs Gene Expressions in Gills

Cytochrome C oxidase (COX) is the last and rate-limiting enzyme in the respiratory electron transport chain in cells and can cause the transmembrane proton electrochemical potential difference that ATP synthase then uses to synthesize ATP, so its activity directly affects the ATP synthesis rate (Keilin and Hartree, 1938). We used “Mytilus” and “Cytochrome C oxidase” as key words to search the gene bank and found 3 common gene sequences, COX I, COX II, and COX III, and 1 particular gene sequence, COX IV. Thus, 4 COX I-IV structural subunits of *M. edulis* were selected as target genes, and their expression profiles were determined by qRT-PCR analysis.

The extraction of total RNA and the synthesis of cDNA were performed according to Wang et al. (2011). The obtained cDNA mix was stored at −80°C and diluted 8-fold for subsequent qRT-PCR analysis.

qRT-PCR analysis was carried out according to Zhang et al. (2008) with the *M. edulis* actin gene as the endogenous control. The mRNA expressions of COXs were analyzed by the 2^−ΔΔCt^ method (Livak and Schmittgen, 2001), and data were expressed in terms of the relative mRNA expressions. All the primers used for qRT-PCR were listed in Table 2.

**Table 2:** Sequences of the primers used in the experiment.

Gene	Accession number	Direction	Primer sequence (5ʹ-3ʹ)
*actin*	AF172606	F	CTCCACTCAATCCAAAAGCC
		R	AACCGTATGCGTGACACCA
*COX I*	GU570522	F	CGTTCTTATTGTGGCTCTTCATT
		R	CTCGCTCCCCTTTTATCTCC
*COX II*	EU332534	F	AGAAAACCCCCAGGATACATT
		R	ACGAATGAAGTACATCCGACCT
*COX III*	U50219	F	GTAACTCAAGCCCATAAGAG
		R	ATGCTCTTCTTGAATATAAGCGTACC
*COX IV*	EU332541	F	AGATTATCCCTTCCCAGCACT
		R	GGCATTCATCTCAGAAAAGGTT

*F, forward primer; R, reverse primer*.

### Statistical Analysis

Data were expressed as means±standard deviation (SD) and analyzed by SPSS 26.0. The data were first tested for normal distribution and homogeneity of variance by Shapiro-Wilk’s test and Levene’s test, respectively. The significance of each difference between groups was assessed by one-way ANOVA followed by a multiple comparison test (S-N-K). Any difference with a value of *p* < 0.05 was considered significant.

## Results

### Energy Changes of *M. edulis* After Acidification Treatments

**Figure 1:**
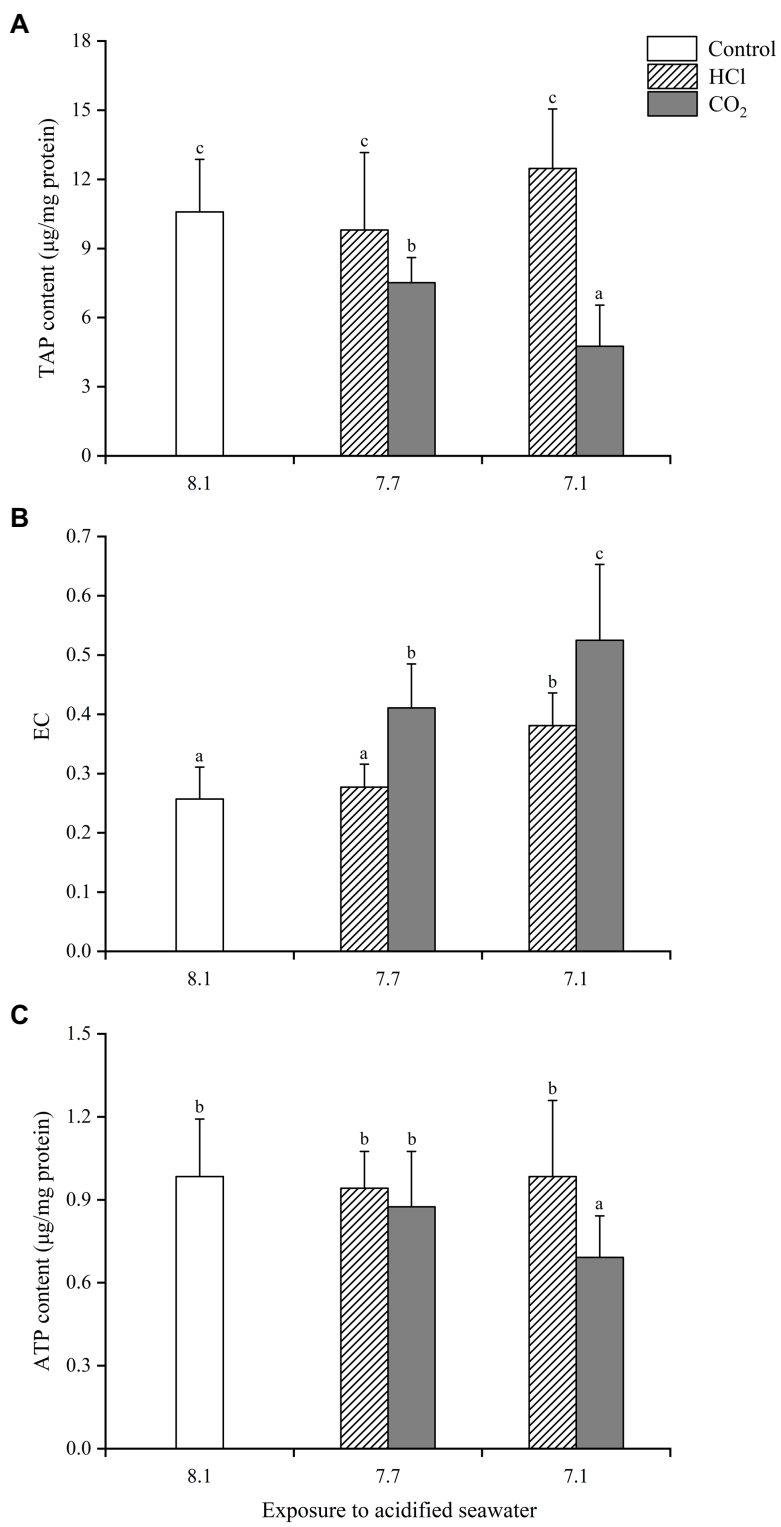
Effects of seawater acidification induced by HCl addition and CO_2_ enrichment on TAP content **(A)**, EC **(B)** and ATP content **(C)** in gills of *Mytilus edulis*. Data were expressed as means±SD (*n*=6). Different letters represent significant differences among experimental groups (*p* < 0.05).

In gills (Figure 1), TAP changed little in either HCl treatment group (*p* > 0.05) compared with the control group; TAP decreased significantly (*p* < 0.05) in both CO_2_ treatment groups compared with the control group, significantly lower at pH 7.1 than pH 7.7. Similarly, ATP remained unchanged in both HCl treatment groups while declined significantly in the CO_2_ treatment group at pH 7.1 (*p* < 0.05). Significant increases of EC (*p* < 0.05) were observed in the HCl treatment group at pH 7.1 and the two CO_2_ treatment groups, significantly higher at pH 7.1 than pH 7.7.

**Figure 2:**
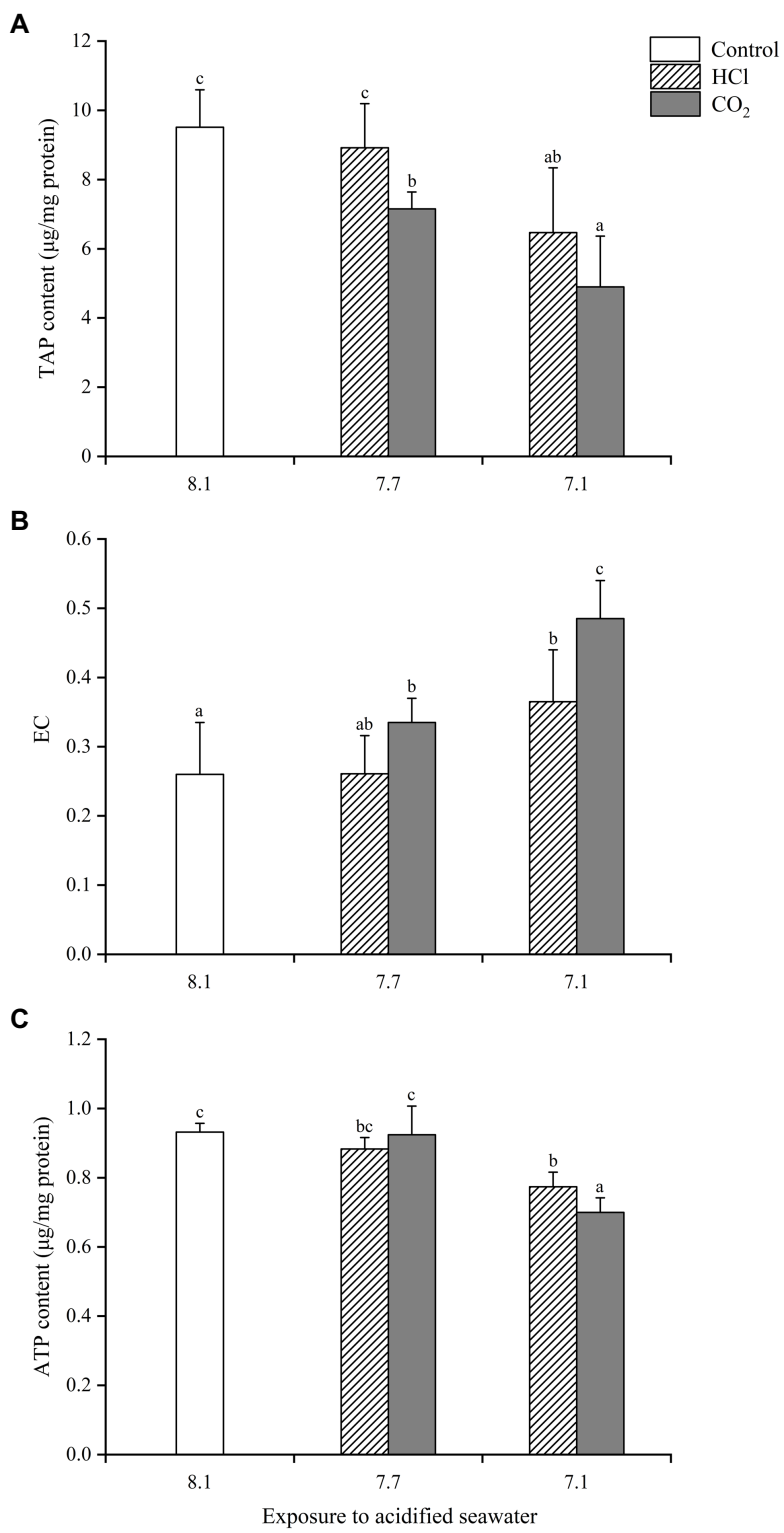
Effects of seawater acidification induced by HCl addition and CO_2_ enrichment on TAP content **(A)**, EC **(B)** and ATP content **(C)** in soft tissue of *M. edulis*. Data were expressed as means±SD (*n*=6). Different letters represent significant differences among experimental groups (*p* < 0.05).

The energy changes in soft tissue were similar to those in gills with some exceptions (Figure 2). For instance, both TAP and ATP in the HCl treatment group at pH 7.1 decreased significantly in soft tissue (*p* < 0.05), while neither changed in gills.

**Figure 3:**
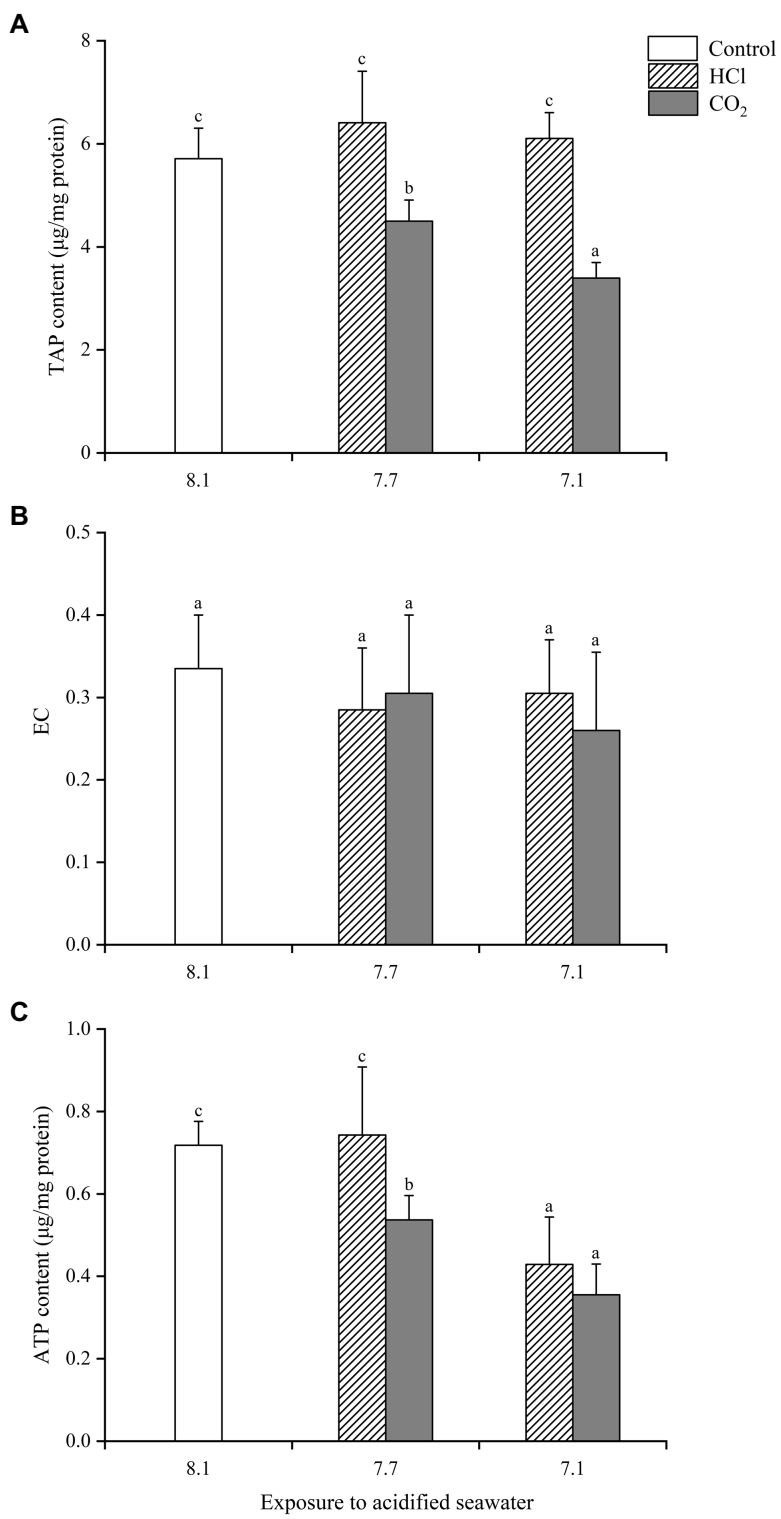
Effects of seawater acidification induced by HCl addition and CO_2_ enrichment on TAP content **(A)**, EC **(B)** and ATP content **(C)** in hemocytes of *M. edulis*. Data were expressed as means±SD (*n*=6). Different letters represent significant differences among experimental groups (*p* < 0.05).

TAP in hemocytes presented similar change trends to those seen in gills (Figure 3). ATP declined significantly in both CO_2_ treatment groups compared with the control group (*p* < 0.05), significantly lower at pH 7.1 than pH 7.7, while it only decreased significantly in the HCl treatment group at pH 7.1 (*p* < 0.05). EC showed no significant change in any treatment group compared with the control group, which partly accounted for the greater decrease in hemocytes ATP after acidification treatments.

In general, the energy changes in the CO_2_ treatment groups were more substantial than those in the HCl treatment groups at the same pH (*p* < 0.05).

**Figure 4:**
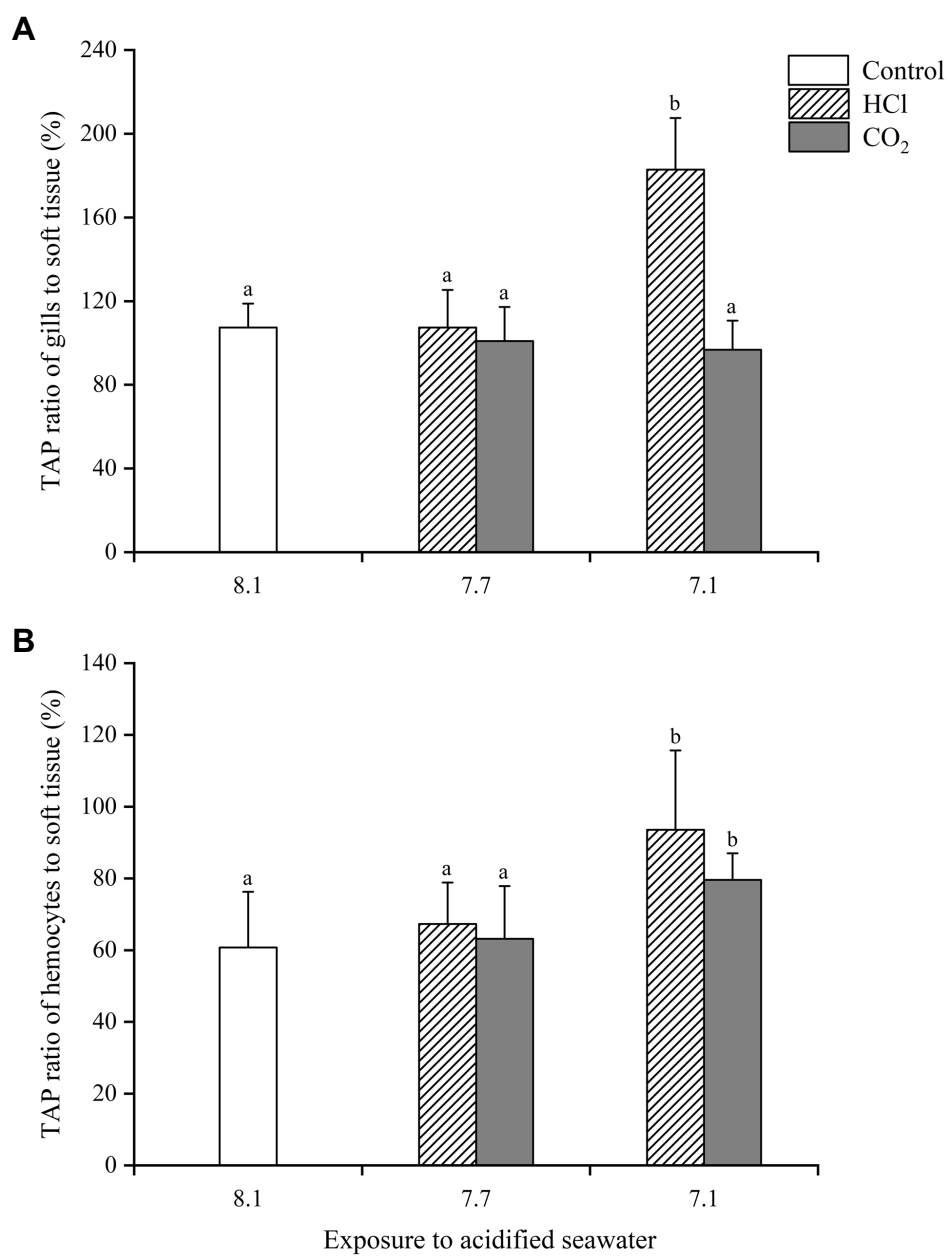
Changes of energy allocation in gills **(A)** and hemocytes **(B)** of *M. edulis* under seawater acidification induced by HCl addition and CO_2_ enrichment. Data were expressed as means±SD (*n*=6). Different letters represent significant differences among experimental groups (*p* < 0.05).

The ratios of the TAP in gills and hemocytes to that in soft tissue were calculated to evaluate the alterations of energy allocation after acidification treatments (Figure 4). At pH 7.7, the TAP ratios of gills and hemocytes changed little in either CO_2_ treatment groups or HCl treatment groups. At pH 7.1, the TAP ratios of gills changed little (*p* > 0.05), while the TAP ratios of hemocytes increased significantly (*p* < 0.05) in the CO_2_ treatment groups; the TAP ratios of both the gills and hemocytes increased significantly (*p* < 0.05) in the HCl treatment groups.

### Expression Profiles of COXs in Gills of *M. edulis* After Acidification Treatments

**Figure 5:**
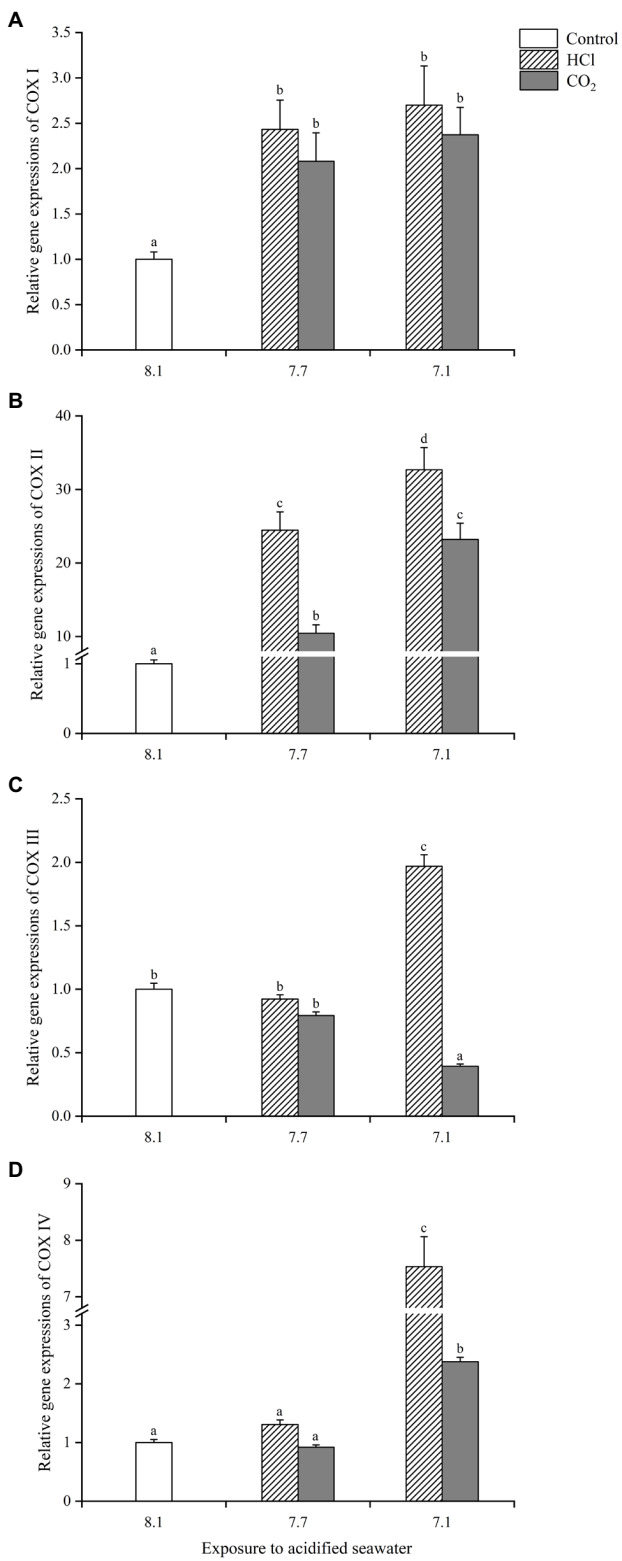
The expression profiles of four cytochrome C oxidases (COXs) [**(A)**: COX I; **(B)**: COX II; **(C)**: COX III; **(D)**: COX IV] in gills of *M. edulis* under seawater acidification induced by HCl addition and CO_2_ enrichment. Data were expressed as means±SD (*n*=6). Different letters represent significant differences among experimental groups (*p* < 0.05).

The 4 COXs in gills analyzed in the present study all responded actively to seawater acidification, but the specific expression profiles varied with different COXs isoforms and acidification methods. The gene expression of COX I was significantly upregulated in each treatment group compared with the control (*p* < 0.05), with the highest upregulation (1.7-fold) observed in the HCl treatment at pH 7.1, but there was no significant difference among the 4 treatment groups (Figure 5A). The gene expression of COX II increased more than 10-fold in each treatment group compared with the control (*p* < 0.05), with the highest upregulation (32.7-fold) observed in the HCl treatment group at pH 7.1; the COX II gene levels were significantly higher at pH 7.1 than pH 7.7 under HCl-induced acidification as well as CO_2_-induced acidification (Figure 5B); moreover, the COX II gene level was significantly higher in the HCl treatment group than the CO_2_ treatment group at the same pH (*p* < 0.05). COX III presented quite different response patterns to CO_2_-induced seawater acidification compared with the other three COXs (Figure 5C). The gene expression of COX III changed little in either treatment group at pH 7.7 (*p* > 0.05); at pH 7.1, it increased significantly in the HCl treatment group (1.97-fold, *p* < 0.05) but decreased significantly (0.39-fold, *p* < 0.05) in the CO_2_ treatment group. The gene expression of COX IV remained unchanged at pH 7.7 (*p* > 0.05) while increased significantly in both treatment groups at pH 7.1 (*p* < 0.05), and the upregulation level (7.5-fold) in the HCl treatment group was significantly higher than that (2.4-fold) in the CO_2_ treatment group (*p* < 0.05; Figure 5D). Of the 4 COXs, COX II was most sensitive to the increase in the seawater acidity, and COX III presented the most different response patterns to the different acidification methods.

## Discussion

Organisms have been reported to adopt a variety of energy strategies to deal with stresses (Lannig et al., 2010; Thomsen and Melzner, 2010; Pan et al., 2015; Liu et al., 2020), and similar observations were obtained in the present study. Energy-related changes were found in *M. edulis* exposed to acidified seawater, including energy reserve (TAP), energy flux (ATP), ATP synthesis rate (EC), energy allocation (TAP ratios), and COXs gene expressions. However, the specific energy changes varied with different pH levels and acidification methods.

The TAP content directly reflects the energy storage status and the ability to generate ATP (Xu et al., 2005). Generally, the TAP content is stable under normal conditions, although the ratios of ATP, ADP, and AMP fluctuate to some extent with different oxidative phosphorylation levels. In the present study, we observed that the TAP contents all decreased significantly (*p* < 0.05) in the three sampled tissues of *M. edulis* exposed to CO_2_-acidified seawater (both pH 7.7 and 7.1 treatments), so was the TAP content in soft tissue of *M. edulis* exposed to HCl-acidified seawater at pH 7.1, which meant that the potential stocks for ATP synthesis decreased. Our previous study reported that seawater acidification damaged the ultrastructure of the gills and digestive glands and prohibited digestive enzyme activities, which ultimately resulted in a decline in feeding and digestion abilities (Xu et al., 2020). We believed that there might be a correlation between the decrease in TAP and the weakening of the assimilation ability.

EC is an effective index indicating the ATP synthesis rate. We observed an elevation of EC in soft tissue but a decrease in ATP content in both HCl and CO_2_ treatment groups at pH 7.1, and similar situation was found in gills in CO_2_ treatment group at pH 7.1. There seemed to be a paradox between the ATP synthesis rate and ATP availability. A reasonable explanation might be that the excessive energy consumption induced by the enhanced acidification was beyond the energy compensation ability and that the equilibrium point between ATP production and consumption could not be reached. Moreover, higher EC values and lower final ATP contents were obtained simultaneously in CO_2_-treated groups than in HCl-treated groups, indicating that more energy expenditure existed in CO_2_-treated groups, which implied that CO_2_ enrichment exerted greater pressure to *M. edulis* than HCl addition (Lannig et al., 2010; Thomsen and Melzner, 2010; Melzner et al., 2020). As gills mainly perform the functions of feeding and breathing, the vitality of the gills is important for the energy-generating processes of *M. edulis* (Doeller et al., 2001). The failure to compensate for the excessive energy consumption of the gills weakened their feeding and repair capabilities, as observed in our previous study (Xu et al., 2020). Hemocytes primarily perform immune functions in mussels (Loker et al., 2004). We found that EC in hemocytes changed little, while the ATP contents in hemocytes decreased significantly in both pH 7.7 (CO_2_) and 7.1 (CO_2_ and HCl) treatment groups compared with the control, which further supported the assumption that seawater acidification, especially induced by CO_2_, resulted in the overconsumption of ATP. We tried to link the ATP content with the filtering rate, ROS production, and phagocytosis, which indicated energy intake, defensive behaviors, and immune function, respectively, and Pearson’s correlation coefficients were thus calculated to present their possible linkages (Sun et al., 2017). The results demonstrated a clear negative correlation between the ATP content and ROS production (*p*=−0.771) and a positive relationship between the ATP content and phagocytosis (*p*=0.732) in the CO_2_-treated groups, but no significant correlation was found in the HCl-treated groups, which suggested that more energy was allocated to resistance to CO_2_ enrichment, and this was a possible explanation for why CO_2_ enrichment presented more detrimental impacts than HCl addition.

Here raised another question, namely, was the alteration of energy allocation necessary for the sustainability of *M. edulis* when facing the stress-induced decrease in energy availability? We attempted to answer this question by calculating the TAP ratios of gills and hemocytes to soft tissue. The results demonstrated that obvious energy reallocation appeared in both CO_2_ and HCl treatments at pH 7.1, but it was more effective in the HCl treatment group than in the CO_2_ treatment group. This result was demonstrated by the fact that the TAP ratios of gills and hemocytes both increased significantly in the HCl group, while only the TAP ratio of hemocytes increased significantly in the CO_2_ group. Several pieces of evidence, including the lower mortality rates and higher growth indexes in HCl groups (Sun et al., 2016, 2017; Xu et al., 2020), have proven the necessity of energy reallocation.

The gene expressions of COXs part in gills generally increased significantly after acidification treatment, meaning a greater potential to accelerate the synthesis rate of ATP, which was consistent with the increasing trend of EC in gills. In fact, as the main rate-limiting enzyme of the mitochondrial respiratory chain, the increase in COXs gene expressions improved the efficiency of the entire respiratory chain, thus providing more energy to the cell (Achard-Joris et al., 2006), which was necessary for gills in a state of high-energy consumption. Liu and Lin (2012) also found that *Pinctada martensii Dunker* upregulated the expression of energy metabolism-related genes to mitigate OA damage. Achard-Joris et al. (2006) suggested that the increased expression of the COX I gene in shellfish exposed to heavy metal pollution contributed to the compensation for the decline in mitochondrial activity and coping with inner oxidative stress. In the present study, the significantly higher gene expressions of the 4 COXs in gills in HCl treatment groups partly accounted for the better maintenance of the ciliary vitality and ingestion capability of its gills (Xu et al., 2020). In summary, the upregulations of the COXs gene expressions were believed to be an active response to seawater acidification at the genetic level.

**Figure 6:**
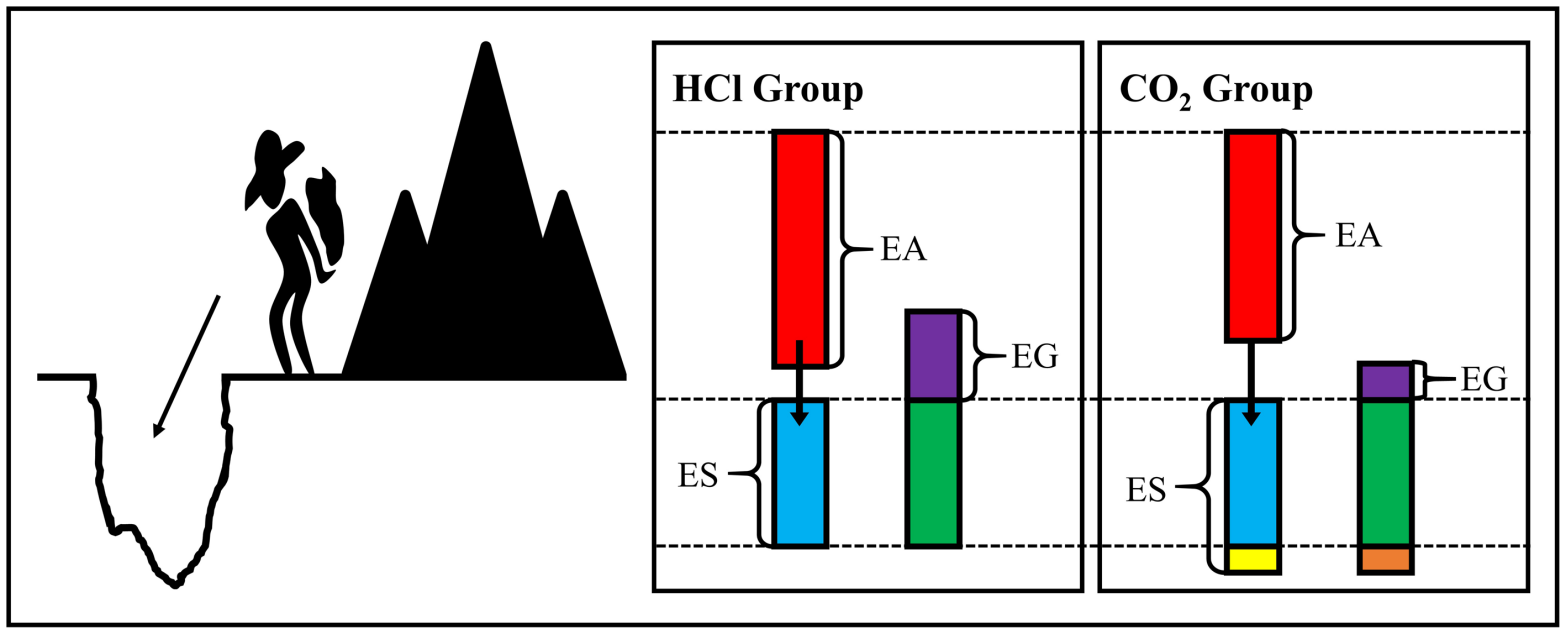
Diagram for “cut and cover hypothesis.” EA, energy availability; ES, energy for survival; EG, energy for growth.

We attempted to propose a hypothesis from the energy perspective, named the “cut and cover hypothesis,” to intuitively explain the growth index differences between the CO_2_ enrichment and HCl addition reported in our previous studies (Figure 6). Specifically, the obtained energy was first used to meet the requirement for the survival “pit” when the organism was exposed to a stressful condition and only the remaining energy was possible for growth, which could be roughly described as “energy availability - energy for survival=energy for growth.” In the present study, the energy availability in the CO_2_-treated group was lower, while the consumed energy for resistance was higher, that is to say, the remaining energy for growth was lower than that in the HCl-treated group. The lower dry weights of the soft tissue and the shell in CO_2_-treated group found in our previous study (Sun et al., 2016) provided favorable evidence for our conjecture.

In the present study, we demonstrated that acidification induced by CO_2_ enrichment was more deleterious than that by HCl addition from the perspective of energy changes. In fact, the mechanisms involved in CO_2_-induced acidification were much more complex than HCl-induced acidification because dissolved CO_2_ affected the biological functions from the molecule to the whole organism not only through H^+^ but also through CO_2_-related changes in the bicarbonate system (Pörtner et al., 2005). In the present study, we found that with increasing H^+^ concentration in CO_2_-treated seawater, the partial CO_2_ pressure (*p*CO_2_), carbonic acid (H_2_CO_3_), and bicarbonate (HCO_3_^−^) concentrations increased, while the carbonate ion concentrations (CO_3_^2−^) and calculated saturation states of aragonite (Ω_Ag_) and calcite (Ω_Cal_) decreased (Table 1), confirming the occurrence of more complex chemical changes in CO_2_-acidified seawater.

*M. edulis* adapted a series of energy strategies, such as increasing the ATP synthesis rate and reallocating more energy to its gills and hemocytes, to cope with seawater acidification and thus had a certain tolerance to moderate seawater acidification. In the present study, by increasing EC and the expressions of COX I/II, the final ATP contents were successfully restored in gills and soft tissues in CO_2_ treatment groups at pH 7.7. However, with the enhancement of acidification, the failure to compensate for the excessive energy consumption was fatal for the survival of *M. edulis*. Meanwhile, CO_2_-induced acidification caused more complex chemical changes in seawater and thus exhibited more deleterious impacts on *M. edulis*. Moreover, owing to the presence of multiple stressors in addition to elevated *p*CO_2_, the challenges are greater for *M. edulis* living under natural OA (Shang et al., 2018, 2020; Gu et al., 2019). If OA continues to intensify, the survival of *M. edulis* will be less likely.

## Data Availability Statement

The raw data supporting the conclusions of this article will be made available by the authors, without undue reservation.

## Author Contributions

YG, BZ, and YW contributed to conception and design of the study. YG, TS, and YJ carried out the experiment. YG, TS, and YZ organized the database and performed the statistical analysis. YG wrote the first draft of the manuscript. BZ wrote sections of the manuscript. All authors contributed to manuscript revision, read, and approved the submitted version.

## Funding

This work was financially supported by the National Nature Science Foundation of China (NSFC) (No. 41776117), the Fundamental Research Funds for the Central Universities (201822005), and the NSFC-Shandong Joint Fund for Marine Ecology and Environmental Sciences (No. U1606404).

## Conflict of Interest

The authors declare that the research was conducted in the absence of any commercial or financial relationships that could be construed as a potential conflict of interest.

## Publisher’s Note

All claims expressed in this article are solely those of the authors and do not necessarily represent those of their affiliated organizations, or those of the publisher, the editors and the reviewers. Any product that may be evaluated in this article, or claim that may be made by its manufacturer, is not guaranteed or endorsed by the publisher.
